# *In vivo* Visualization of Pig Vagus Nerve “Vagotopy” Using Ultrasound

**DOI:** 10.3389/fnins.2021.676680

**Published:** 2021-11-25

**Authors:** Megan L. Settell, Aaron C. Skubal, Rex C. H. Chen, Maïsha Kasole, Bruce E. Knudsen, Evan N. Nicolai, Chengwu Huang, Chenyun Zhou, James K. Trevathan, Aniruddha Upadhye, Chaitanya Kolluru, Andrew J. Shoffstall, Justin C. Williams, Aaron J. Suminski, Warren M. Grill, Nicole A. Pelot, Shigao Chen, Kip A. Ludwig

**Affiliations:** ^1^Department of Biomedical Engineering, University of Wisconsin-Madison, Madison, WI, United States; ^2^Wisconsin Institute of Neuroengineering (WITNe), University of Wisconsin-Madison, Madison, WI, United States; ^3^Mayo Clinic Graduate School of Biomedical Sciences, Mayo Clinic, Rochester, MN, United States; ^4^Department of Radiology, Mayo Clinic, Rochester, MN, United States; ^5^Department of Ultrasound, West China Hospital of Sichuan University, Chengdu, China; ^6^Department of Biomedical Engineering, Case Western Reserve University, Cleveland, OH, United States; ^7^Louis Stokes Cleveland VA Medical Center, Cleveland, OH, United States; ^8^Department of Neurosurgery, University of Wisconsin-Madison, Madison, WI, United States; ^9^Department of Biomedical Engineering, Duke University, Durham, NC, United States; ^10^Department of Electrical and Computer Engineering, Duke University, Durham, NC, United States; ^11^Department of Neurobiology, Duke University, Durham, NC, United States; ^12^Department of Neurosurgery, Duke University, Durham, NC, United States

**Keywords:** vagotopy, histology, vagus nerve, vagus nerve stimulation, bioelectronic medicine, electroceutical, neuromodulation, ultrasound

## Abstract

**Background:** Placement of the clinical vagus nerve stimulating cuff is a standard surgical procedure based on anatomical landmarks, with limited patient specificity in terms of fascicular organization or vagal anatomy. As such, the therapeutic effects are generally limited by unwanted side effects of neck muscle contractions, demonstrated by previous studies to result from stimulation of (1) motor fibers near the cuff in the superior laryngeal and (2) motor fibers within the cuff projecting to the recurrent laryngeal.

**Objective:** Conventional non-invasive ultrasound, where the transducer is placed on the surface of the skin, has been previously used to visualize the vagus with respect to other landmarks such as the carotid and internal jugular vein. However, it lacks sufficient resolution to provide details about the vagus fascicular organization, or detail about smaller neural structures such as the recurrent and superior laryngeal branch responsible for therapy limiting side effects. Here, we characterize the use of ultrasound with the transducer placed in the surgical pocket to improve resolution without adding significant additional risk to the surgical procedure in the pig model.

**Methods:** Ultrasound images were obtained from a point of known functional organization at the nodose ganglia to the point of placement of stimulating electrodes within the surgical window. Naïve volunteers with minimal training were then asked to use these ultrasound videos to trace afferent groupings of fascicles from the nodose to their location within the surgical window where a stimulating cuff would normally be placed. Volunteers were asked to select a location for epineural electrode placement away from the fascicles containing efferent motor nerves responsible for therapy limiting side effects. 2-D and 3-D reconstructions of the ultrasound were directly compared to *post-mortem* histology in the same animals.

**Results:** High-resolution ultrasound from the surgical pocket enabled 2-D and 3-D reconstruction of the cervical vagus and surrounding structures that accurately depicted the functional vagotopy of the pig vagus nerve as confirmed via histology. Although resolution was not sufficient to match specific fascicles between ultrasound and histology 1 to 1, it was sufficient to trace fascicle groupings from a point of known functional organization at the nodose ganglia to their locations within the surgical window at stimulating electrode placement. Naïve volunteers were able place an electrode proximal to the sensory afferent grouping of fascicles and away from the motor nerve efferent grouping of fascicles in each subject (*n* = 3).

**Conclusion:** The surgical pocket itself provides a unique opportunity to obtain higher resolution ultrasound images of neural targets responsible for intended therapeutic effect and limiting off-target effects. We demonstrate the increase in resolution is sufficient to aid patient-specific electrode placement to optimize outcomes. This simple technique could be easily adopted for multiple neuromodulation targets to better understand how patient specific anatomy impacts functional outcomes.

## Introduction

The therapeutic effects of vagus nerve stimulation (VNS) for epilepsy and heart failure, while significant in some patients, are often limited by intolerable side effects including throat tightening or pain, voice changes, hoarseness, cough, and dyspnea (Morris and Mueller, 1999; Howland, 2014). The inadvertent stimulation of somatic nerve branches extending from the vagus, such as the superior and recurrent laryngeal nerve (SLN and RLN, respectively), has been implicated as the cause of these side effects (Tosato et al., 2007; Yoo et al., 2013; Nicolai et al., 2020). These nerve branches are either activated through stimulation of fascicles within the stimulating cuff (RLN), or by current escaping the cuff (SLN) (Boon et al., 2009; Castoro et al., 2011; Nicolai et al., 2020). The SLN and RLN innervate neck muscles involved in many of the therapy-limiting side effects and therefore avoiding stimulation of these nerve fibers is paramount.

The vagus nerve (VN) contains a topographical organization (Settell et al., 2020), or vagotopy, that has the potential to be visualized using ultrasound. Previous work in a pig model of VNS demonstrated a bimodal functional organization in the VN. In the nodose ganglia (NG), pseudo-unipolar cell bodies (predominately sensory afferents) are grouped into a large fascicle, distinct from a separate, smaller grouping of nerve fibers. This secondary grouping of nerve fibers gives rise to the superior and recurrent laryngeal nerve branches (Settell et al., 2020). This bimodal arrangement of fascicles could be used to strategically place VNS cuffs to avoid the neuronal projections that innervate muscles implicated in side effects. Current clinical VNS cuffs wrap approximately 270° around the vagus nerve, and thus stimulate the circumference of the trunk mostly indiscriminately. Strategic placement of small electrodes and utilization of a current steering stimulation protocol, to target sensory over motor regions, could minimize therapy-limiting activation of the neck muscles, and optimize clinical efficacy.

Visualization of peripheral nerves using ultrasound could be an effective intraoperative method to identify fascicular organization and pertinent anatomical information *in vivo.* Ultrasound offers higher resolution, and is more cost-effective than other imaging modalities such as magnetic resonance imaging (MRI) (Zaidman et al., 2013). The use of ultrasound for neuropathology was first reported in the 1980s, with improvements in capabilities over the last thirty years (Cartwright et al., 2017). Non-invasive ultrasound has been completed in patients on a variety of superficial nerves demonstrating fascicular resolution. The sciatic nerve has been visualized in patients using ultrasound during popliteal sciatic nerve block for hallux valgus surgery (bunionectomy), with clear visualization of the epineurium through the skin (Karmakar et al., 2013). The median, radial and ulnar nerves, are more superficial than the sciatic nerve and have been visualized through the skin during carpal tunnel evaluation with slightly better resolution of fascicles (Marciniak et al., 2013; Taylor et al., 2016). In 2016, the FDA approved a high frequency ultrasound device for human-use, which further improved imaging of superficial nerve fascicles such as those in the median nerve (Cartwright et al., 2017).

Despite the ability to visualize these superficial nerves, visualizing fascicular organization of the VN with ultrasound poses a unique problem, as it is below several layers of skin, fat, and muscle. Current capabilities of the clinical transducers do not allow for high-resolution, non-invasive visualization of the fascicular organization of deep nerves such as the VN (Brown et al., 2016; Inamura et al., 2017; Ottaviani et al., 2020). Though non-invasive ultrasound of the VN has been established in the clinical setting for diagnosis of masses of the neck (Giovagnorio and Martinoli, 2001), the depth of penetration is not sufficient to observe fascicular organization, and resolution tends to be poor (Inamura et al., 2017). In humans, the VN is 36.2 ± 9.4 mm (mean ± SD) from the surface of the skin, with no differences between sides or sexes (Hammer et al., 2018). Given the depth of the VN, we propose a novel approach for visualizing vagotopy by placing the ultrasound transducer within the surgical pocket to improve resolution without increasing surgical risk.

Here, we demonstrate this simple intraoperative methodology for visualization of the vagotopy of the pig VN using a high frequency (50 MHz) ultrasound transducer within the surgical pocket. We characterize the utility of ultrasound placed within the surgical pocket to (1) identify the bimodal organization between the pseudo-unipolar cell bodies (sensory afferents) and the secondary fascicle grouping giving rise to the SLN and RLN at the level of the nodose ganglia, (2) resolve the bimodal organization of fascicles within the surgical window, and (3) obtain additional information about the fascicular organization of the SLN and RLN themselves that may be useful in seeding computational models to inform off-target activation. Ultrasound images at selected locations were compared to histological images to confirm underlying vagotopy.

To test the utility of this information for aiding in surgical placement of epineural electrodes, ultrasound images were provided to a group of volunteers, with minimal training, who were asked to follow the sensory afferents from the pseudo-unipolar cell plane to a region where the VNS cuff is usually placed. Volunteers were asked to place the center of the stimulating contact as far from the motor efferents as possible at the specified VNS cuff location, within the surgical window. Thus, demonstrating the feasibility of using ultrasound to optimize contact placement near sensory afferents intended for therapy and to prevent off-target, therapy-limiting side effects (motor-efferents). These results demonstrate that real-time ultrasound can be collected, analyzed, and used to inform electrode cuff placement. This simple approach could lead to patient-specific, optimized placement of implanted electrodes for a variety of neuromodulation targets, resulting in reduced effects on off-target fibers and potentially more efficacious stimulation.

## Materials and Methods

### Subjects

All study procedures were approved by the University of Wisconsin—Madison and Mayo Clinic Institutional Animal Care and Use Committee. Additionally, procedures completed at the Mayo Clinic were conducted under the guidelines of the American Association for Laboratory Animal Science in accordance with the National Institutes of Health Guidelines for Animal Research (Guide for the Care and Use of Laboratory Animals). Subjects included 3 healthy domestic (Yorkshire/Landrace crossbreed) swine (2F/1M; mean ± SD = 41 ± 1.71 kg). All subjects were housed individually (21°C and 45% humidity) with ad libitum access to water and were fed twice a day. Each subject was given an intramuscular injectable induction anesthesia: telazol (6 mg/kg), xylazine (2 mg/kg), and glycopyrrolate (0.006 mg/kg). An intramuscular injection of buprenorphine was given as an analgesic (0.03 mg/kg). A blood pressure catheter was placed in the femoral artery (Millar, Inc., Houston, TX, Model # SPR-350S), and an intravenous catheter placed in the peripheral ear vein for drug and fluid administration. Subjects were endotracheally intubated and maintained with a mechanical ventilator using 1.5–3% isoflurane. All vital signs including temperature, heart rate, CO2, and respiration were continuously collected and recorded every 15 minutes and used to monitor depth of anesthesia. To aid in the quantification of fascicular structure, an additional set of 3 swine (2F/1M; mean ± SD = 33 ± 14.7 kg were scanned post-mortem, and will hereby be referred to as cadaver studies. These subjects underwent all surgical and ultrasound methods as listed below unless otherwise noted.

### Surgical Methods

The surgical approach for exposing the VN and microdissection procedures have been described previously (Settell et al., 2020). Briefly, in a dorsal recumbence position, a ventral incision was made on the subject’s right side, just lateral and parallel to midline starting at the level of the mandible. Tissue was divided to locate the carotid sheath which was incised to expose the carotid artery, internal jugular vein, and VN. The VN was bluntly dissected from the nodose ganglion to approximately 10 cm caudal; careful measures were taken to avoid disturbing any of the surrounding branches, such as the SL or sympathetic trunk (ST). This exposed region spans the equivalent location for cervical VNS implantation in a patient, as identified by a practicing neurosurgeon (Nicolai et al., 2020; Settell et al., 2020). The incision site was kept moist with 0.9% sterile saline until the completion of experiment. In the additional cadaver experiments the surgical pocket was extended cranially to expose the superior cervical ganglion (SCG) to locate the branching of the ST.

### Ultrasound

**FIGURE 1:**
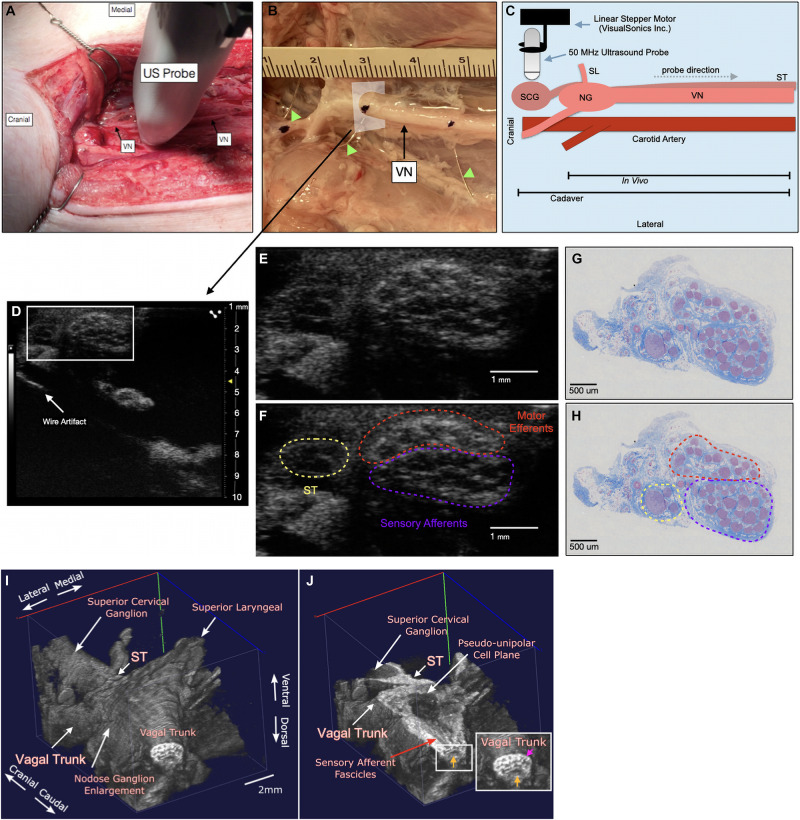
Ultrasound method for visualizing the vagus nerve in a representative subject **(A)** the swine surgical window includes the right vagus nerve (VN) and the carotid artery, as well as the 50 MHz ultrasound probe (US probe) moving in the cranial to caudal direction. Skin, muscle, and fat were retracted in this acute preparation. **(B)** Representative VN in one of the cadaver subjects showing fiducial wire (green arrow heads) and histology dye as well as cross section of US plane **(D)**. **(C)** Schematic of the probe direction (gray dashed arrow) as it scanned from the nodose ganglion (NG) (*in vivo*) or superior cervical ganglion (SCG) (cadaver) moving caudally, approximately 10 cm. **(D)** Ultrasound cross section demonstrating bimodal fascicular organization within the surgical window, with wire fiducial marker and zoom of nerve indicated with white box **(E,F)**. **(E,F)** Zoomed region of ultrasound still (white box, **D**) demonstrating sensory and motor bimodal arrangement (purple and red regions, respectively) as traced from the pseudo-unipolar cell plane of the nodose ganglion (See Supplementary Video, Cadaver Subject 1 for the full ultrasound video) The sympathetic trunk (ST), as traced from the SCG is indicated in yellow **(G,H)** A representative, paired histology section (5 μm, Gomori’s trichrome) showing the bimodal organization of the vagus nerve within the surgical window, at the wire fiducial marker, indicating the bimodal sensory and motor fascicular organization, as indicated by the purple and red regions, respectively. The ST is indicated in yellow. **(I)** Subject 1 3D ultrasound data clearly showing the nodose ganglion and superior laryngeal extending ventromedially, and vagal trunk extending in the cranial and caudal direction. The superior cervical ganglion is just cranial to the nodose ganglion, with the sympathetic trunk (ST) running parallel to the vagus nerve along the dorsomedial aspect. **(J)** Coronal plane (ventral to dorsal) of vagus nerve showing axons extending from the pseudo-unipolar cell plane to the sensory afferent mode of the vagal trunk. In the zoomed region of the vagal trunk, sensory afferent fascicles are indicated by the yellow arrow, and motor efferent fascicles are indicated by the pink arrow.

The ultrasound approach for this study was described previously (Huang et al., 2019). Briefly, after the surgical procedure, all ultrasound images were collected using a Vevo^®^ 3100 (live) or Vevo^®^ 2100 (cadaver) high frequency imaging system (FUJIFILM VisualSonics Inc., Toronto, Canada). The high frequency 50 MHz linear array transducer (MX700, 35 μm nominal axial resolution, 70 μm nominal lateral resolution, 9 mm × 10 mm imaging window) was placed within the surgical pocket (Figure 1A), 1–2 mm above the VN to obtain axial cross sections (Figures 1B,C). The surgical pocket was filled with mineral oil (live subjects) or room temperature saline (cadaver experiments, live to cadaver comparison subject) to increase coupling between the transducer and nerve, and the vagus nerve suspended from surrounding tissue using vessel loops to limit movement artifact and improve image quality. The transducer was attached to a linear stepper motor (P/N 11484, VisualSonics Inc.) connected to the Vevo^®^ integrated rail system to allow for smooth acquisition of images along the length of the nerve, without the need for manual manipulation. The transducer was directed to move along the length of the VN in the cranial to caudal direction, starting at the nodose ganglion or SCG (Figure 1C) and extending the length of the surgical window (approximately 10–12 cm in length), with image collection including the typical VNS cuff location. 3-D plane-by-plane volumetric B-mode images were collected (Figures 1D–F). Data associated with this study (Settell et al., 2021), were collected as part of the Stimulating Peripheral Activity to Relieve Conditions (SPARC) program and are available through the SPARC Portal (RRID: SCR_017041) under a CC-BY 4.0 license.

To aid in the confirmation of sensory afferent vs. motor efferent fascicles in ultrasound data we used a system of wire fiducials paired with histology dye to indicate specific regions along the vagus nerve; (1) SCG (2) NG (3) the region of cervical vagus nerve (cVN) where the clinical stimulating cuff is placed (Figure 1B). Wire fiducials placed underneath the vagus nerve created artifacts in the ultrasound images, allowing us to directly pair these ultrasound images with their corresponding histology slices as indicated by histology dye (Figures 1B,D–H). Additionally, 3-D reconstructions of ultrasound data were created (Figures 1I,J, see Ultrasound Video Analysis for Methods).

### Histology and Microdissection

At the completion of ultrasound scanning, the VN was exposed further to identify clearly branches extending from the main trunk, including the ST which courses parallel to the VN, and the RLN bifurcation at the level of the subclavian artery. Connective tissue was removed, and histological dye was placed along the lateral and ventral edges of the vagus nerve to maintain orientation information (Bradley Products, Inc., Davidson Marking System, Bloomington, MN).

The VN was then excised from just cranial to the nodose ganglion to the RLN bifurcation (*in vivo*) or from just cranial to the SCG to the cVN (cadaver). The vagus nerves were placed in 10% neutral buffered formalin for approximately 48 h at 4°C. Samples were then placed in a Research and Manufacturing Paraffin Tissue Processor (RMC Ventana Renaissance PTP 1530, Ventana Medical Systems, Oro Valley, AZ), and they underwent a series of standard processing steps to dehydrate, clear, and infiltrate with paraffin wax (see Settell et al., 2020 for details). Embedded samples were sectioned at 5 μm, mounted on charged slides, and stained using Gomori’s trichrome. Slides were imaged at 20x using a Zeiss Axio Imager 2 with a Zeiss digital camera (Figures 1G,H).

### Ultrasound Video Analysis

To provide quantification of the ultrasound data to evaluate its utility in tracking sensory afferent fascicles from the nodose ganglia to the region of stimulation, we obtained data in an additional three cadavers. We then created a set of tutorial videos to train naïve ultrasound users on how to identify key markers in the ultrasound video; (1) SCG, (2) NG (as identified by pseudo-unipolar cell plane), (3) motor efferent fascicles, (4) SLN, and the (5) sensory afferents projecting from the pseudo-unipolar cell plane of the nodose ganglia (Supplementary Tutorial Videos 1–3). Volunteers were then instructed on how to trace the grouping of sensory fibers from the NG to the region of the stimulating cuff (as noted by the wire artifact). Once volunteers felt comfortable with the process, they were asked to make an attempt at placing a hypothetical stimulating contact on the remaining two subject’s ultrasound videos they had not previously viewed. The only guidance provided in the remaining two videos was an analog clock face placed over the nerve at the correct cervical level, so volunteers could provide a time to indicate their selected location (Supplementary Test Videos 1–3). To confirm whether volunteers successfully located sensory vs. afferent grouping, histological slices corresponding to the wire fiducials for the selected location were compared for each subject. This blinded process allowed us to evaluate the feasibility of the technique for aiding in identifying the specific locations of fascicles, using both modalities. Additionally, data was converted into 3-D volumetric videos (Supplementary 3D Cadaver Videos). Ultrasound images were exported in B-mode from the Vevolab software and imported into Fiji to convert them into v3draw format. Images were then converted to 3-D data using vaa3D (Peng et al., 2010, 2014a,b).

## Results

### Ultrasound of the Vagus Nerve to Identify Key Anatomical Features

**FIGURE 2:**
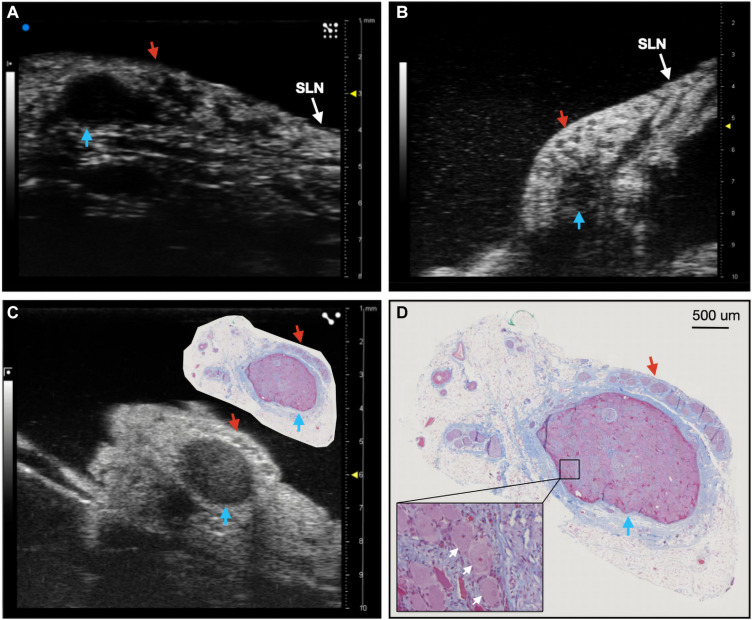
Ultrasound of the nodose ganglia in two live, and one cadaver subject (*n* = 3). Blue arrows indicate the hypoechoic pseudo-unipolar cell region of the nodose ganglia, red arrows indicate the motor efferent region fascicles. **(A,B)** Ultrasound of the nodose ganglia and superior laryngeal nerve (SLN) *in vivo.* **(C)** Ultrasound of the nodose ganglion in a cadaver with paired histology slice demonstrating the pseudo-unipolar cell region **(D)** Histology slice at a larger scale with zoomed region of pseudo-unipolar cells (white arrows) and surrounding satellite cells.

Ultrasound videos were of sufficient resolution to generate 3-D reconstructions suitable to identify key features at a place of known functional organization at the nodose ganglia, and trace associated fascicles to their location at the stimulating cuff region (Figure 1I). The pseudo-unipolar cells of the nodose were identified via ultrasound as a single large fascicle, or large circular hypoechoic region within the nodose ganglia (Figure 2). The region of fascicles above the pseudo-unipolar cell region have previously been traced to the superior laryngeal and recurrent laryngeal in histology, and are putatively mainly motor and parasympathetic efferent fibers (Settell et al., 2020). Therefore, using ultrasound we were able to trace the afferent fascicles arising from the pseudo-unipolar cell groupings, beginning at the nodose ganglia into the cervical region of the vagus nerve. This bimodal organization, while previously shown in histology, could be visualized at various points along the length of the cervical VN [see Supplementary Material for the full 3D ultrasound videos, *n* = 3 (live), and *n* = 3 (cadaver)].

**FIGURE 3:**
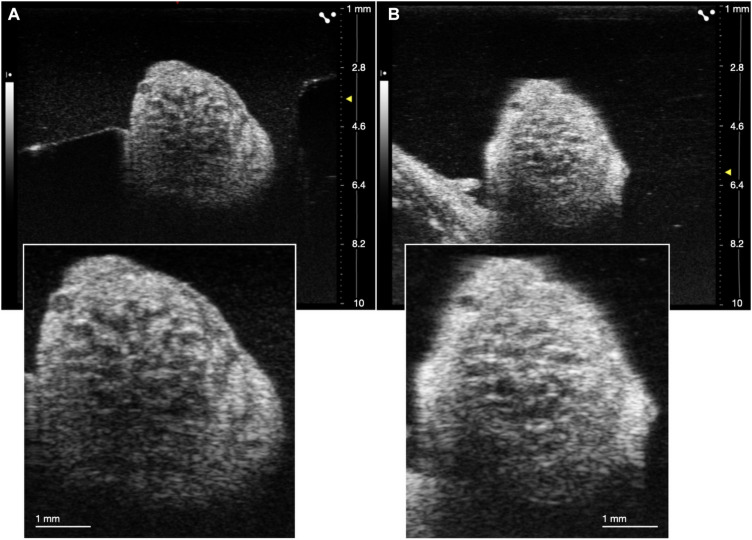
Comparison of ultrasound in live **(A)** and cadaver **(B)** swine. Insets are demonstrating clear visualization of fascicles in both states.

Despite visualization of fascicular structure, further quantification and evaluation of this technique for its utility in clinical applications was warranted. We repeated this approach in cadaver swine to allow for the placement of wire fiducials in an expanded surgical pocket (*n* = 3). To ensure that fascicular structure and organization in both live and cadaver models was clear and easily identifiable, we directly compared the vagus nerve of one subject both pre and *post-mortem* (Figure 3).

### Ultrasound of the Superior and Recurrent Laryngeal Branches

**FIGURE 4:**
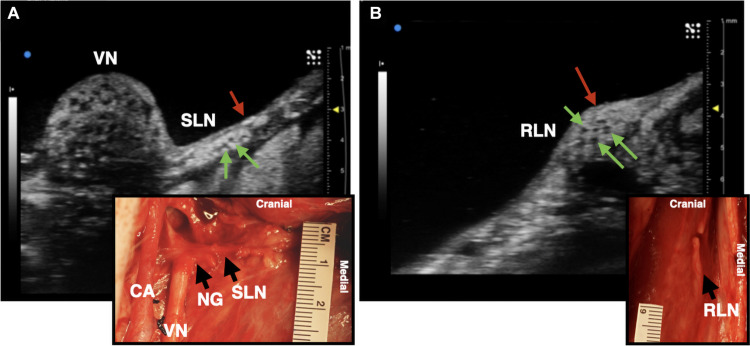
Ultrasound images of the superior laryngeal nerve (SLN) and recurrent laryngeal nerve (RLN) branches of the vagus nerve (VN) in one live representative subject (*n* = 1). **(A)** The SLN (red arrow) branching ventromedially off of the nodose ganglion (NG). Green arrows indicate fascicles within the nerve. **(B)** The RLN (red arrow), running along the esophageal groove. Green arrows indicate fascicles. Photograph insets in both **(A,B)** depict the corresponding ultrasound region within the surgical pocket; carotid artery (CA).

We assessed whether ultrasound could be used during the surgical procedure to visualize the RLN and SLN branches of the vagus nerve, as these are implicated in off-target activation of the deep neck muscles that produce therapy-limiting side effects (Nicolai et al., 2020). Despite these branches being smaller in diameter than the compound VN, we were able to locate both within the surgical window both visually and using ultrasound, with clear visualization of fascicular structure. The SLN extends ventromedially from the NG to innervate the cricoarytenoid (internal branch of the superior laryngeal) and cricothyroid (external branch of the superior laryngeal) muscles of the throat (Figure 4A; Hayes et al., 2013; Settell et al., 2020). The RLN was identified as running parallel to the vagus nerve along the esophagus and inserting into the cricoarytenoid muscle. It contained far fewer fascicles, but was clearly visible (Figure 4B).

### Quantification of Fascicular Organization Using Volunteers

**FIGURE 5:**
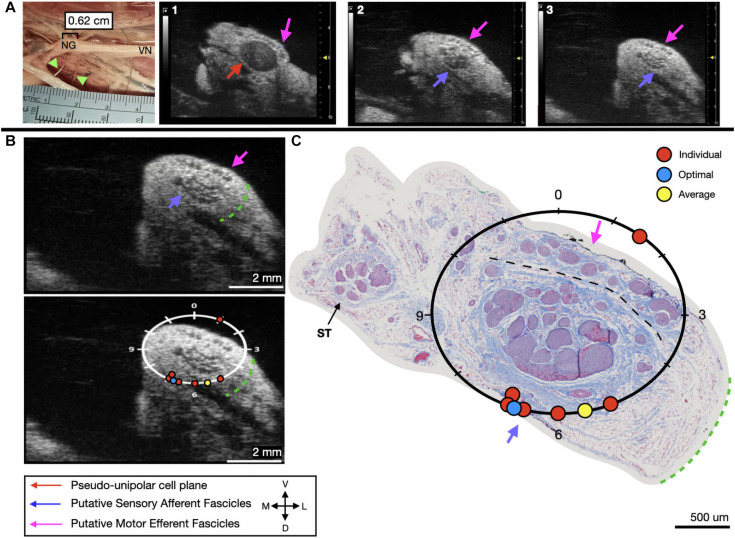
Subject 1 **(A)** From Left to Right: Location of wire fiducials within the surgical pocket. (Note, this subject’s surgical photo does not contain histology dye, however, it was placed on the nerve before removal from the pocket). The location of the paired ultrasound video and required placement of stimulating contact is at wire fiducial two [0.62 cm from the superior laryngeal coming off of the nodose ganglion (NG)]. **(A1–3)** Progressive ultrasound images in the cranial to caudal direction from the NG to the cervical vagus nerve (VN), and location where volunteers were requested to place the stimulating contact (1–3, respectively). Red arrow indicates pseudo-unipolar cell region of the NG, pink arrows indicate motor efferent region, purple arrow indicates sensory afferent region, as confirmed via histology. **(B)** Ultrasound cross sections of wire fiducial two, where volunteers were asked to trace the sensory afferent axons from the NG. Top panel: pink arrows note motor efferent region, purple arrow indicates the sensory afferent fascicle grouping, green dashed line indicates area of transected connective tissue during removal for histology. Bottom panel: analog clock face placed on test video to give volunteers locations to place the hypothetical stimulating contact based on their tracing task, green dashed line indicates area of transected connective tissue during removal for histology. **(C)** Histology slice from wire fiducial two, as indicated with histology dye, with analog clock face to demonstrate stimulating contact locations as placed by volunteers in the ultrasound video. Green dashed line indicates area of transected connective tissue during removal for histology. **(B,C)** Red circles indicate each volunteer’s placement of the stimulating contact, the yellow circle indicates the average response, and the blue circle indicates the optimal contact location. Sympathetic trunk (ST), medial (M), ventral (V), dorsal (D), lateral (L).

**FIGURE 6:**
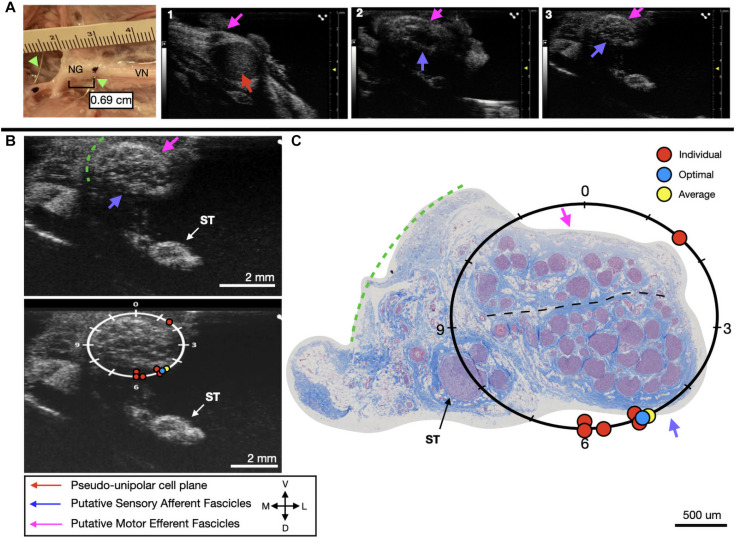
Subject 2 **(A)** From Left to Right: Location of wire fiducials and histology dye within the surgical pocket. The location of the paired ultrasound video and required placement of stimulating contact is at wire fiducial two [0.69 cm from the superior laryngeal coming off of the nodose ganglion (NG)]. **(A1–3)** Progressive ultrasound images in the cranial to caudal direction from the NG to the cervical vagus nerve (VN), and location where volunteers were requested to place the stimulating contact (1–3, respectively). Red arrow indicates pseudo-unipolar cell region of the NG, pink arrows indicate motor efferent region, purple arrow indicates sensory afferent region, as confirmed via histology. **(B)** Ultrasound cross sections of wire fiducial two, where volunteers were asked to trace the sensory afferent axons from the NG. Top panel: pink arrows note motor efferent region, purple arrow indicates the sensory afferent fascicle grouping, green dashed line indicates area of transected connective tissue during removal for histology. Bottom panel: analog clock face placed on test video to give volunteers locations to place the hypothetical stimulating contact based on their tracing task, green dashed line indicates area of transected connective tissue during removal for histology. **(C)** Histology slice from wire fiducial two, as indicated with histology dye, with analog clock face to demonstrate stimulating contact locations as placed by volunteers in the ultrasound video. Green dashed line indicates area of transected connective tissue during removal for histology. **(B,C)** Red circles indicate each volunteer’s placement of the stimulating contact, the yellow circle indicates the average response, and the blue circle indicates the optimal contact location. Sympathetic trunk (ST), medial (M), ventral (V), lateral (L), dorsal (D).

We next sought to determine if volunteers with minimal prior training could trace the axonal projections from the pseudo-unipolar cell plane of the nodose ganglia into the cervical region of the vagus nerve, where the stimulating cuff would traditionally be placed from an ultrasound video to which they were naïve (Table 1). In subject 1 and 2, five of six volunteers were able to successfully trace the sensory afferent region from the nodose ganglia (Figures 5A, 6A); with the average contact placed well within the sensory afferent region as identified via histology, opposite the motor efferent grouping of fascicles (Figures 5B,C, 6B,C). Electrode placements were largely consistent across evaluators in these two subjects, with one clear outlier, likely placed in error, on the opposite mode. As can be seen in Figures 5, 6 the placement by the remaining evaluators was tightly clustered around the ideal placement point as identified by histology.

**TABLE 1:** Responses for the location of the stimulating contact from each volunteer, based on the subject videos provided.

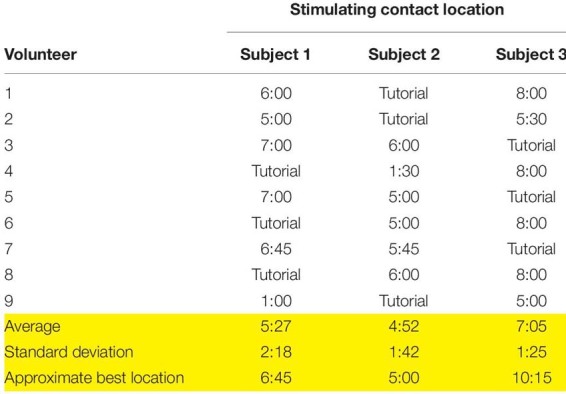

*Stimulating contact location refers to the analog clock face placed over the vagus nerve in the ultrasound video (see Supplementary Tutorial and Test Videos). Average responses are given with standard deviation for each of the three subjects, along with the approximate best location as determined by histology (yellow rows).*

**FIGURE 7:**
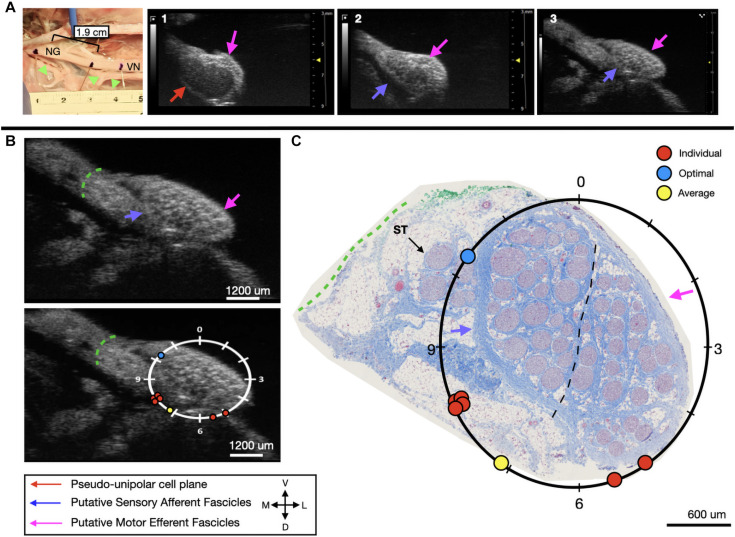
Subject 3 **(A)** From Left to Right: Location of wire fiducials and histology dye within the surgical pocket. The location of the paired ultrasound video and required placement of stimulating contact is at wire fiducial two [1.9 cm from the superior laryngeal coming off of the nodose ganglion (NG)]. **(A1–3)** Progressive ultrasound images in the cranial to caudal direction from the NG to the cervical vagus nerve (VN), and location where volunteers were requested to place the stimulating contact (1–3, respectively). Red arrow indicates pseudo-unipolar cell region of the NG, pink arrows indicate motor efferent region, purple arrow indicates sensory afferent region, as confirmed via histology. **(B)** Ultrasound cross sections of wire fiducial two, where volunteers were asked to trace the sensory afferent axons from the NG. Top panel: pink arrows note motor efferent region, purple arrow indicates the sensory afferent fascicle grouping, green dashed line indicates area of transected connective tissue during removal for histology. Bottom panel: analog clock face placed on test video to give volunteers locations to place the hypothetical stimulating contact based on their tracing task, green dashed line indicates area of transected connective tissue during removal for histology. **(C)** Histology slice from wire fiducial two, as indicated with histology dye, with analog clock face to demonstrate stimulating contact locations as placed by volunteers in the ultrasound video. Green dashed line indicates area of transected connective tissue during removal for histology. **(B,C)** Red circles indicate each volunteer’s placement of the stimulating contact, the yellow circle indicates the average response, and the blue circle indicates the optimal contact location. Sympathetic trunk (ST), medial (M), ventral (V), lateral (L), dorsal (D).

In the third subject, there was an extensive amount of undissected fat and connective tissue, as can be seen in the ultrasound video, that was subsequently dissected to perform histology. This additional tissue would make it problematic to place an electrode on the epineural surface at the approximate mid-point of the sensory mode close to these fascicles. Therefore, despite the “optimal placement” (10:15) being distant from the group (8:00), the most accessible placement *in vivo*, would be closer to 7 o’clock. Four out of six volunteers were able to correctly place the stimulating cuff for subject three, with these placements clustered at the only realistic location an electrode could be placed (Figure 7A). Across all six evaluators the average placement was still within the sensory afferent region of the nerve (Figures 7B,C).

## Discussion

### Toward Improving Intraoperative Placement of Vagus Nerve Stimulation Cuffs

Surgical implantation of VNS devices has limited patient specificity (Reid, 1990; Terry et al., 1990, 1991). Briefly, in the current clinical surgical method, the carotid sheath is located medial to the muscle and undergoes blunt dissection and is opened approximately 7 cm to expose the carotid artery, internal jugular vein, and VN. Vessel loops are used to suspend the VN while approximately 3 cm are dissected from any surrounding tissue to allow for proper placement of cuff electrodes. Three helical cuffs are then placed around the nerve (two stimulating electrodes and an anchor) (Giordano et al., 2017). The simple and widely deployable introduction of ultrasound into this VNS implantation process could significantly aid in identifying (1) fascicular organization of the VN, (2) branches extending from the VN implicated in producing side effects, and (3) optimized locations for cuff placement based on patient-specific anatomy.

Using anatomical landmarks, ultrasound is effective for clinical evaluation of superficial somatic peripheral nerves (Lawande et al., 2014) and has greater sensitivity for detection of neuropathologies than MRI (Zaidman et al., 2013). The median nerve can be consistently visualized from the mid-upper arm to the wrist using high frequency, linear-array transducers (Brown et al., 2016). *Post-mortem* visualization of the RLN via ultrasound is used in studying neuropathologies such as vocal cord paralysis (Solbiati et al., 1985). Ultrasound has also been used clinically for detection of pathologies in peripheral nerves such as tumors and leprosy (Martinoli et al., 2000). Non-invasive imaging of the VN has been conducted both in patients (Park et al., 2011) and cadavers (Knappertz et al., 1998), with visualization of the carotid artery, jugular vein, and VN. However, resolution tends to be poor and the only visually obvious components tend to be the jugular vein and carotid artery, with the VN difficult to identify (Knappertz et al., 1998). There has been significant work in creating a database of ultrasound images of the VN to provide neurosurgeons with a resource for predicting the location of the VN and the distribution of the depths of the nerve from the skin’s surface (Inamura et al., 2017). Though the use of ultrasound in this manner highlights the ability to view the VN non-invasively in relation to the carotid artery and jugular vein, it also demonstrates the poor resolution for viewing fascicular structure, and other pertinent branches (external branch of the SLN, RLN). Current literature suggests that resolution is just clear enough to visualize nerves based on surrounding anatomical landmarks, such as the internal jugular vein for identification of the vagus nerve; and quantitative measurements are usually in the form of cross-sectional area (Curcean et al., 2020; Horsager et al., 2021). Thus, there is a clear gap in datasets for understanding fascicular organization as it pertains to clinical stimulation. Here we address this, by placing the high frequency transducer within the surgical pocket and utilizing the increased resolution to determine fascicular organization based on known anatomical landmarks such as the nodose ganglion.

We used a pig VNS model to validate the concept of using high frequency ultrasound within the surgical pocket, to improve resolution. The ultrasound transducer was placed in the surgical pocket of anesthetized pigs that were undergoing VNS experiments. The skin incision in the pig model (10–12 cm) is slightly larger than that of the human preparation (∼7 cm), and the skin, fat, and muscle were retracted in the animal model to optimize transducer placement. The cavity was filled with mineral oil (*in vivo, n* = 3) or saline (*in vivo, n* = 1; cadaver, *n* = 3) to improve coupling to the nerve. The VN was visible in the ultrasound with clear, identifying, features in both the live and cadaver models. From the ultrasound images, we visualized the fascicular organization with sufficient resolution to identify the pseudo-unipolar cell region of the nodose ganglion (Figure 2), the bimodal organization (Figures 5–7), and the SLN and RLN branches (Figure 4). When these images were compared to *post-mortem* histology, it was determined that this approach is not only easily deployable during the procedure but captures the anatomical organization in real-time. Volunteers, not practiced in reading ultrasound were able to visualize the organization of the vagus nerve, based on a single training video. This suggests that in the clinical setting, this technique could be very useful in the initial placement of the stimulating cuff to avoid motor efferent fibers and limit off-target effects. This information could also be used to inform patient programming at future clinical visits. Finally, fascicular variance from subject to subject, or even within subject, may play a key role in therapeutic efficacy; data obtained via ultrasound intraoperatively could be used to assess the relationship between responder/non-responder and subject specific fascicular organization.

### Avoiding Off-Target Effects by Identifying Off-Target Nerves

The SLN and RLN are implicated in many of the off-target effects of VNS (Nicolai et al., 2020). We aimed to evaluate the utility of ultrasound as a tool for visualizing the SLN and RLN within the surgical pocket, and identify fascicular organization. As compared to the pig model, the human SLN—which branches at the level of the nodose (inferior ganglion)—may be more difficult to discern, as the nodose ganglion is typically cranial to the surgical window, and therefore simply tracing the vagus nerve back to its point of origination is not feasible. Though the SLN is smaller and contains fewer fascicles than the vagal trunk, ultrasound could potentially be used as a quick confirmation for identifying the nerve within the surgical window (Figure 4A), and for seeding computational models to inform off-target activation. As the SLN innervates several muscles of the neck that are implicated in side effects of VNS (Yoo et al., 2013; Nicolai et al., 2020), it is imperative that intraoperative placement of the VNS cuff not be in a region where current escape could activate the SLN resulting in off-target activation.

The anatomy of the SLN can vary between patients (Whitfield et al., 2010). Injuries to the external branch of the superior laryngeal (ESL) nerve, which innervates the cricothyroid muscle, result in voice changes, a common side effect of VNS (Whitfield et al., 2010). The classic anatomy of the ESL, and its relationship to traditional landmarks such as the superior thyroid artery or superior pole of the thyroid, is highly variable (Whitfield et al., 2010). Before placing the VNS cuff, the use of ultrasound to identify the ESL, which extends into the surgical window, could aid in minimizing some of the off-target effects that occur. Data from this study demonstrate visualization of the VN, SLN, and RLN can be achieved through imaging within the surgical pocket to store 3-D reconstructions for future analyses.

Our study demonstrates the degree to which ultrasound information within the surgical window could be personalized, not only in terms of VN location, and fascicular organization, but the location of surrounding structures. A patient-specific surgical approach, tailored by ultrasound, would allow the surgeon to consider variations in vagal branching and location, or potential variances in vagal fascicular orientation. Adding the ultrasound component to the current surgical approach, would not only aid in patient-specific cuff placement, but introduces minimal risk, as the time needed to scan the nerve is minimal (minutes) once the surgical area is prepared. Additionally, patient-specific ultrasound images could inform computational models of VNS. Computational models are critical for the development and application of neurostimulation devices, specifically in terms of optimizing the post-surgical programming process. Individualized models, seeded by patient-specific fascicular organization obtained from ultrasound could increase the speed and process of programming, and may be critical for practically programming multi-contact electrode designs in the future. Existing models for non-invasive VNS are based on high-resolution MRI and focus solely on the activation of specific targeted fiber types (Mourdoukoutas et al., 2018). However, it has been shown that ultrasound imaging provides greater resolution and sensitivity than MRI for peripheral nerves (Zaidman et al., 2013).

Future computational models should consider off-target activation for better quantitative predictions of the potential side effects of VN activation. Greater consideration must be given to the SLN and RLN in future models for VNS, which can be achieved through visualizing vagotopy and the region surrounding the implant using ultrasound. Current three dimensional MRI and finite element-based models of compound peripheral nerves incorporate realistic geometries, as well as inhomogeneous and anisotropic electrical properties of specific nerve elements such as the perineurium and endoneurium (Mourdoukoutas et al., 2018; Pelot et al., 2018). In the future, existing finite element modeling can be used to develop more realistic VN models through consideration of VN fascicular structure, gathered from ultrasound images.

Additionally, this work highlights the opportunity for improved electrode design. Clinical VNS cuffs currently stimulate a large portion of the nerve (270°), therefore despite improved placement to avoid motor efferents, electrodes may still activate unwanted regions. Future electrode designs may include smaller, multi-contact electrodes that encompass all 360°, allowing for clinicians to stimulate differing pairs of contacts, driven by patient-specific imaging data, to improve patient outcomes.

### Limitations

There are several limitations to this study that should be taken into consideration. While the pig VN is similar in size to that of the human VN (Settell et al., 2020), it is at a different depth and requires a different surgical approach. The pig surgical window contains much more fat and muscle than typical human necks and therefore requires more retraction. The retracted surgical preparation allowed for the placement of the ultrasound transducer directly above the nerve (1–2 mm), something that may need to be modified in the clinical setting. Additionally, the cadaver subjects underwent a more extensive surgical opening, allowing for imaging more cranial than in a normal preparation. Furthermore, connective tissue was removed, and the nerve was positioned perpendicular to the ultrasound probe to obtain clear images. This may be more difficult in the clinical setting as care is taken to disrupt the nerve as little as possible, however, given the length of nerve exposed for cuff placement, orientation should not be as much of a barrier. Given the fascicle size in the human vagus is on average larger than the pig, presumably making them easier to resolve via ultrasound, this advantage in humans may offset some of the aforementioned limitations (Pelot et al., 2020; Settell et al., 2020).

A slight difference in resolution of fascicles was noted between a few subjects, independent of live or *post-mortem* state, and is most likely attributed to the acoustic impedance of tissue (temperature, water content, blood flow etc.), or potentially the amount of connective tissue surrounding the nerve. However, in both states, fascicles were clearly identifiable and motor efferent and sensory afferent groupings could be traced into the cVN. As the state of the vagus nerve effects acoustic impedance, future studies involving formalin fixed human cadavers should consider effects on resolution (Sawhney et al., 2017).

In addition to variations in anatomy, the process of preparing the histology may cause the nerve to shrink (Stickland, 1975), which may affect the appearance of the histology, despite being paired to ultrasound via wire fiducials and histology dye. However, the overall appearance of fascicles in the high resolution ultrasound was clear enough that the vagotopy was visible throughout both modalities.

Furthermore, the nodose ganglion in humans is located near the base of the skull in the jugular foramen, more cranial from the surgical window than in a pig model. However, the hypoechoic region of pseudo-unipolar cells is quite large in pigs and could potentially be identified in humans either non-invasively (pre- or intra-operatively) or by aiming the transducer toward the ganglion. This could allow identification of the bimodal organization and subsequent tracking to the surgical window. The feasibility of the translation of this imaging method from pigs to humans may be evaluated in cadavers.

## Conclusion

Vagus nerve stimulation is FDA-approved for several indications, including epilepsy and depression, and holds promise for many other indications. However, for improved clinical VNS efficacy, fascicular organization of the VN should be considered for each patient. Ultrasound is an established method for visualization of these characteristics in somatic nerves and could be implemented during the surgical implantation of the VNS lead to inform placement of cuff electrodes and to inform patient-specific computational models.

Our findings demonstrated the ability to identify the vagotopy of the pig VN intraoperatively with a high-resolution transducer placed in the surgical pocket. We identified the pseudo-unipolar cell aggregation of the nodose ganglia and were able to visualize bimodal organization of fascicular bundles through the cervical trunk where a VNS electrode would be placed. Our subset of cadaver ultrasound data were paired with *post-mortem* histology to confirm fascicular organization, and the technique verified by a set of naïve volunteers. This work highlights the potential for an intraoperative technique that could improve VNS cuff placement, aid in limiting unwanted side effects, and therefore hold promise for enabling patient-specific computational models to inform stimulation paradigms. The simple method of using the surgical pocket to place the ultrasound transducer closer to the nerve target of interest, without increasing patient risk, could also be readily applied to numerous other neuromodulation therapies.

## Data Availability Statement

The raw data supporting the conclusions of this article will be made available by the authors, without undue reservation.

## Ethics Statement

The animal studies were reviewed and approved by the Mayo Clinic and University of Wisconsin-Madison Institutional Animal Care and Use Committees.

## Author Contributions

MS: conceptualization, methodology, data curation, writing—original draft and edited draft, and data analysis. AS: methodology, data curation, writing—original draft and edited draft, and data analysis. RC: methodology, data curation, writing—edited draft, and data analysis. MK: writing—original draft and data analysis. BK, CH, CZ, AU, and CK: methodology, data curation, writing—original draft, and data analysis. EN: conceptualization, methodology, and data curation. JT: methodology, writing, edited draft, and data analysis, AJSh and AJSu: conceptualization, methodology, data curation, writing—original draft, and data analysis. JW: conceptualization, funding acquisition, and writing—original draft. WG and NP: conceptualization, data analysis, writing—original draft. SC: methodology, data curation, writing—original draft, and data analysis. KL: conceptualization, methodology, funding acquisition, supervision, writing—original draft and edited draft, and data analyses.

## Conflict of Interest

JW and KL were scientific board members and have stock interests in NeuroOne Medical Inc., a company developing next generation epilepsy monitoring devices. JW also has an equity interest in NeuroNexus technology Inc., a company that supplies electrophysiology equipment and multichannel probes to the neuroscience research community. KL was also a paid member of the scientific advisory board of Cala Health, Blackfynn, Abbott and Battelle. KL was also a paid consultant for Galvani and Boston Scientific. KL was a consultant to and co-founder of Neuronoff Inc. None of these associations are directly relevant to the work presented in this manuscript.

The remaining authors declare that the research was conducted in the absence of any commercial or financial relationships that could be construed as a potential conflict of interest.

## Publisher’s Note

All claims expressed in this article are solely those of the authors and do not necessarily represent those of their affiliated organizations, or those of the publisher, the editors and the reviewers. Any product that may be evaluated in this article, or claim that may be made by its manufacturer, is not guaranteed or endorsed by the publisher.
