# Supplementary material for: In vivo Visualization of Pig Vagus Nerve “Vagotopy” Using Ultrasound
Source: Front Neurosci. 2021 Nov 25;15:676680. doi: 10.3389/fnins.2021.676680 (PMC8660563; doi:10.3389/fnins.2021.676680)
Supplement: Supplementary file 13 [file Image_1.pdf]

Figure Caption for Video 1.mp4:
3D Ultrasound of Cadaver Subject 1

Figure Caption for Video 2.mp4:
3D Ultrasound of Cadaver Subject 2

Figure Caption for Video 3.mp4:
3D Ultrasound of Cadaver Subject 3

Figure Caption for Video 4.mp4:
3D Ultrasound of Live Subject 1

Figure Caption for Video 5.mp4:
3D Ultrasound of Live Subject 2

Figure Caption for Video 6.mp4:
3D Ultrasound of Live Subject 3

Figure Caption for Video 7.mp4:
Subject 1 Test Ultrasound Video

Figure Caption for Video 8.mp4:
Subject 2 Test Ultrasound Video

Figure Caption for Video 9.mp4:
Subject 3 Test Ultrasound Video

Figure Caption for Video 10.mp4:
Subject 1 Tutorial Ultrasound Video

Figure Caption for Video 11.mp4:
Subject 3 Tutorial Ultrasound Video

Figure Caption for Video 12.mp4:
Subject 2 Tutorial Ultrasound Video
